# Epidemiology and treatment of surgical infections after distal radius fractures: a systematic review

**DOI:** 10.1007/s00402-025-06061-x

**Published:** 2025-10-23

**Authors:** Andrea Cruciani, Cristina Giuli, Giulia Di Pietro, Luigi Cianni, Camillo Fulchignoni, Pierluigi Del Vecchio, Giulio Maccauro, Raffaele Vitiello

**Affiliations:** 1 Department of Orthopaedics and Traumatology, Università Cattolica del Sacro Cuore, 00168 Rome, Italy; 2 Department of Medical and Surgical Sciences, Unit of Infectious Diseases, Fondazione Policlinico Universitario A. Gemelli IRCCS, 00168 Rome, Italy; 3 Department of Orthopaedics and Traumatology, Fondazione Policlinico Universitario A. Gemelli IRCCS, 00168 Rome, Italy; 4 Department of Orthopaedics and Traumatology, Unit of Hand Surgery, Fondazione Policlinico Universitario A. Gemelli IRCCS, 00168 Rome, Italy

**Keywords:** Distal radius fracture, External fixators, Fracture-related infection, Open reduction and internal fixation, Pin-tract infection, Surgical site infection

## Abstract

**Introduction:**

Distal radius fractures (DRFs) are among the most frequent injuries treated by orthopaedic surgeons. Although postoperative infection is uncommon, it represents a clinically relevant complication that may affect outcomes. This systematic review aimed to evaluate the incidence, subtypes, and treatment of infections following surgical management of DRFs.

**Materials and methods:**

A systematic search of MEDLINE/PubMed and Cochrane Library was performed from inception to June 2024, following PRISMA guidelines. English-language longitudinal studies (prospective or retrospective) reporting infection after DRF surgery were included. Case reports, meta-analyses, animal studies, and articles without relevant outcomes were excluded. Extracted data included infection incidence, classification, microbiological findings, and reported management.

**Results:**

Fifty-five studies met inclusion criteria, encompassing 6499 patients and 6451 procedures. A total of 341 infections were reported (5.3%). Superficial surgical site infections accounted for 22.6% of cases, deep infections for 12.0%, and pin-tract infections for 61.0%. Infection rates differed by fixation method: approximately 2.0% for open reduction and internal fixation, 12.0% for Kirschner-wire fixation, and 13.9% for external fixation. Microbiological confirmation was reported only in a minority of studies; when available, *Staphylococcus aureus* was the most frequently isolated organism. Management strategies ranged from oral antibiotics and local wound care for superficial infections to intravenous antibiotics with debridement and hardware removal for severe cases.

**Conclusions:**

Infections following DRF surgery are relatively rare but vary across fixation techniques, with pin-tract infections predominating in percutaneous and external constructs. The heterogeneity of infection definitions and the scarcity of microbiological reporting limit comparability between studies. Standardized SSI/FRI classification and more consistent documentation are needed to improve evidence-based prevention and treatment strategies.

**Supplementary Information:**

The online version contains supplementary material available at 10.1007/s00402-025-06061-x.

## Introduction

Distal radius fractures (DRFs) are among the most common skeletal injuries treated by orthopaedic surgeons, with a characteristic bimodal distribution in young, high-energy trauma patients and older individuals with osteoporosis [1–5]. Advances in surgical fixation have improved outcomes in unstable or displaced fractures, yet complications remain possible. Infection, although relatively infrequent, may delay bone healing, necessitate revision procedures, and negatively affect functional recovery [6, 7].

The reported incidence of infection after DRF fixation varies widely across studies, largely due to heterogeneous definitions and inconsistent reporting [8–12]. Superficial and deep surgical site infections (SSIs) are often grouped together, while pin-tract infections related to percutaneous or external fixation are inconsistently classified. Recent consensus definitions emphasize the importance of distinguishing surgical site infection from fracture-related infection (FRI), particularly in the presence of implants [7].

Previous systematic reviews have mainly focused on functional outcomes and complication rates after DRF fixation, with infection usually reported only as a secondary endpoint [13–17]. To date, no systematic review has comprehensively synthesized the incidence, classification, microbiology, and treatment of infections specifically after DRF fixation.

The objective of this systematic review was therefore to collate and summarize the available evidence on the epidemiology and management of surgical infections after DRF surgery, stratified by fixation technique, in order to identify patterns and inform clinical practice [8].

## Materials and methods

### Search strategy

This systematic review was conducted in accordance with the Preferred Reporting Items for Systematic Reviews and Meta-Analyses (PRISMA) guidelines [18]. A comprehensive literature search of PubMed/MEDLINE and the Cochrane Library was performed from database inception to June 30, 2024. Both Medical Subject Headings (MeSH) and free-text keywords were used, combined with Boolean operators. The complete search strategies for each database are provided in Appendix 1. No filters for publication date, study size, or article type were applied. Grey literature, preprints, and non-indexed sources were not considered. Reference lists of included studies were manually screened to identify additional relevant articles.

### Study selection

Eligible studies were prospective or retrospective longitudinal studies published in English that reported infection after surgical treatment of distal radius fractures. Case reports, meta-analyses, animal studies, and studies unrelated to surgical fixation or infection outcomes were excluded.

Three reviewers independently screened all titles and abstracts, followed by full-text evaluation of potentially relevant papers. Disagreements were resolved by consensus or by a senior author. The methodological quality of included studies was assessed using the Methodological Index for Non-Randomized Studies (MINORS) score [19].

### Data extraction and analysis

Infections were classified according to CDC 2017 criteria as superficial or deep surgical site infections (SSI). Infections occurring up to 12 months in the presence of implants were additionally considered fracture-related infections (FRIs), in line with recent consensus definitions [7]. Pin-tract infections related to external fixators or percutaneous K-wires were recorded separately and not classified as CDC-defined SSI.

Extracted data included: author(s), year of publication, study design, number of patients, fracture type (open/closed, AO classification), mean age, follow-up duration, treatment modality (ORIF, external fixation, Kirschner-wire fixation, prosthesis), infection type and timing, identified pathogens, and management strategies (conservative or surgical).

All data were extracted into a standardized spreadsheet and verified by two senior authors. Results were synthesized descriptively; no meta-analysis or inferential statistical testing was performed.

## Results

### Search and literature selection

**Fig. 1:**
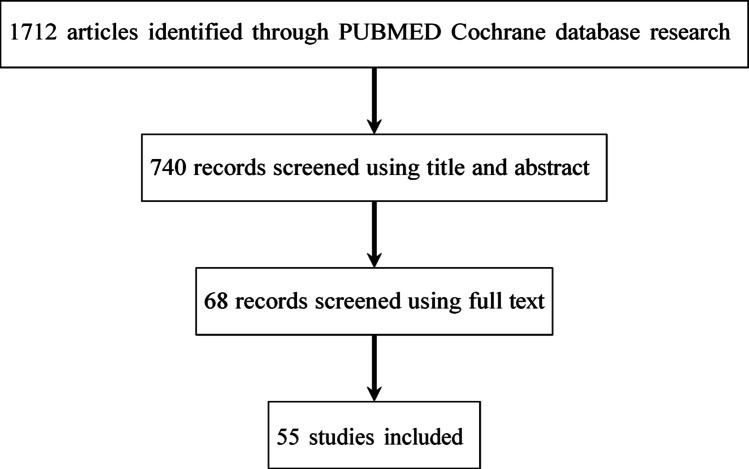
Search and literature selection

The initial database search yielded 1712 records. After removal of duplicates and screening by title and abstract, 68 full-text articles were assessed for eligibility. Of these, 13 were excluded because they did not provide infection-related outcomes, were limited to case reports or very small series, presented overlapping cohorts with other included studies, were published in non-English language, or lacked sufficient details on surgical management. Ultimately, 55 studies fulfilled the inclusion criteria and were included in the final analysis, comprising a total of 6499 patients (Fig. 1).

### Study characteristics

A total of 55 studies were included in this systematic review. Of these, 37 studies (67.3%) were retrospective, and 18 (32.7%) were prospective in design. A total of 6499 patients (2834 male and 3665 female) who underwent 6451 procedures for the treatment of DRFs were evaluated. The reported mean patient age across studies ranged from 21 to 89 years; the median of study-level means was 49.6 years. In two studies [20, 21], the mean age was not reported. The male-to-female distribution was 43.61% and 56.39%, respectively. The mean follow-up duration was 14.5 months, with study-level means ranging from 3 to 60 months.

The characteristics of the included studies are summarized in Table 1.

**Table 1:** Demographic data

Ref.	Year of publication	Manuscript category	No patients	Age (mean)	No male	No female
Kong et al. [8]	2024	Retrospective	474	48.9	247	227
Akdemir et al. [22]	2024	Retrospective	164	53.7	65	99
Huang et al. [20]	2023	Retrospective	95	NR	33	62
Crook et al. [23]	2023	Retrospective	531	46	268	263
Wasiak et al. [24]	2023	Prospective	116	11	75	41
Kotsalis et al. [25]	2023	Retrospective	67	48	33	34
Abdullah et al. [26]	2023	Prospective	15	36.55	14	1
Eckstein et al. [27]	2023	Retrospective	55	81.5	6	49
Moutinot et al. [28]	2022	Retrospective	158	89	11	147
Meng et al. [9]	2022	Retrospective	930	48	482	448
Zhu et al. [29]	2022	Retrospective	423	46	244	179
Meng et al. [30]	2022	Retrospective	78	37	47	31
Zhong et al. [31]	2022	Retrospective	46	54	19	27
Chung et al. [32]	2019	Retrospective	296	68	133	163
Ficke et al. [21]	2018	Retrospective	47	NR	33	14
Jose et al. [33]	2017	Retrospective	53	39.12	42	11
Khatri et al. [34]	2016	Retrospective	23	32.82	17	6
Natoli et al. [35]	2016	Retrospective	16	46.1	8	8
Chilakamary et al. [36]	2016	Prospective	26	47	15	11
Mirghasemi et al. [37]	2015	Prospective	54	53.53	19	35
Matullo et al. [38]	2015	Retrospective	20	48	12	8
Herzberg et al. [39]	2018	Retrospective	25	77	1	24
Özkan et al. [40]	2018	Retrospective	34	47	6	28
Takada et al. [41]	2012	Retrospective	87	41	26	61
Tarallo et al. [42]	2013	Retrospective	303	56	118	185
Kaufman et al. [43]	2013	Retrospective	21	69.5	6	15
Gogna et al. [44]	2013	Prospective	27	30.12	22	5
Wei et al. [45]	2014	Retrospective	22	65	7	15
Lauder et al. [46]	2015	Retrospective	18	61	13	5
Das et al. [47]	2011	Prospective	32	41.4	18	14
Kurylo et al. [48]	2011	Retrospective	32	59	16	16
Lakshmanan et al. [49]	2010	Retrospective	43	49	13	30
Wick et al. [50]	2009	Retrospective	67	61.4	22	45
Tyllianakis et al. [51]	2010	Retrospective	20	53.5	7	13
Herdrich et al. [52]	2009	Prospective	60	56	34	26
Zettl et al. [53]	2009	Prospective	120	66	30	90
Glueck et al. [54]	2009	Retrospective	42	35	27	15
Pieske et al. [55]	2008	Prospective	80	51.62	13	67
Strohm et al. [56]	2007	Retrospective	93	59	42	51
Egol et al. [10]	2006	Prospective	118	54	43	75
Wong et al. [57]	2005	Prospective	30	58.6	11	19
Lee et al. [58]	2003	Prospective	22	44.6	11	11
Ahlborg et al. [59]	1999	Retrospective	310	68	66	244
Chin et al. [60]	1999	Prospective	4	21	4	0
Fritz et al. [61]	2022	Retrospective	61	50	48	13
Lundqvist et al. [62]	2022	Prospective	147	61	30	117
Ahmad et al. [63]	2022	Retrospective	190	48	90	100
Michael et al. [64]	2022	Prospective	32	39.8	21	11
Liechti et al. [65]	2021	Prospective	28	58.1	12	16
Henry et al. [66]	2022	Prospective	24	63	6	18
Gaibor et al. [67]	2020	Retrospective	16	46.5	3	13
Maleitzke et al. [68]	2020	Retrospective	176	59.9	57	119
Maradei-Pereira et al. [69]	2021	Prospective	211	54.5	56	155
Tarallo et al. [70]	2020	Retrospective	110	58	33	77
Liu et al. [71]	2020	Retrospective	207	58	99	108

### Classification and treatment methods of distal radius fractures (DRFs)

**Fig. 2:**
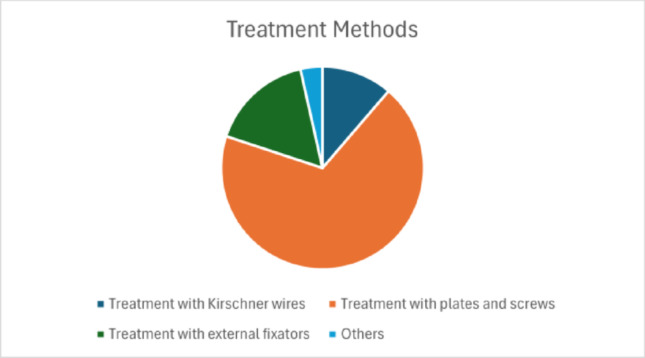
Treatment methods

Most DRFs in the included studies were intra-articular. According to the AO classification, 1,484 were classified as type A, 1,043 as type B, and 2,908 as type C fractures. Among them, 228 were open fractures. According to the Gustilo-Anderson classification [72], 118 were type I, 64 were type II, and 31 were type III. The most commonly used treatment was internal fixation with plates and screws (68.8%), followed by external fixation (16.4%) and Kirschner-wire fixation (11.3%) (Fig. 2).

### Infection rate

Infections were classified according to CDC 2017 criteria. Superficial SSIs were defined as infections involving skin or subcutaneous tissue within 30 days of surgery, while deep SSIs involved deeper soft tissues and could occur up to 90 days postoperatively [11]. Infections occurring later in the presence of implants were considered fracture-related infections (FRIs), according to international consensus [7, 73]. Pin-tract infections related to percutaneous K-wires or external fixators were recorded separately.

**Fig. 3:**
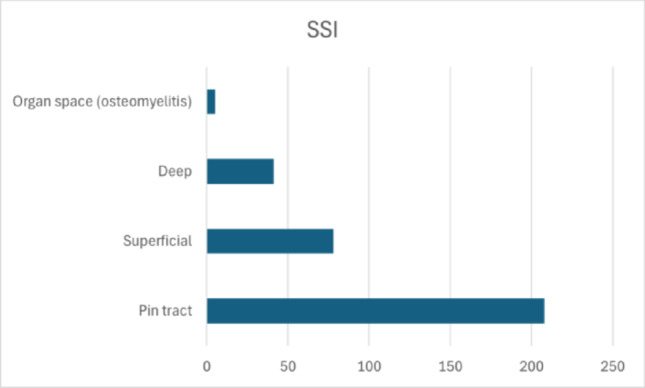
Distribution of surgical site infection

Of the 6451 procedures reviewed, 341 (5.3%) were complicated by infection. Among these, 77 were superficial (22.6%), 41 deep (12.0%), and 208 pin-tract infections (61.0%). Five cases of osteomyelitis (1.5%) were reported. In 10 cases, the authors reported an infection without specifying the subtype (Fig. 3)

Among the 341 infections, 199 were successfully treated with oral antibiotics and local wound care. In 71 cases, intravenous antibiotics and surgical reintervention were required. Surgical management included wound revision with debridement and, in most cases, removal of the fixation hardware. In selected cases, implants were temporarily retained until fracture union was achieved. Nine patients developed fracture malunion.

By treatment group, Kirschner wire fixation was associated with an infection rate of 12.2% (89/728), ORIF with plates and screws with 2.05% (91/4,438), and external fixation with 13.88% (147/1059). Prosthetic implants were used in 27 cases, with no postoperative infections reported. In 199 patients managed with combined treatment (e.g., temporary external fixation followed by ORIF, or simultaneous ORIF and retained K-wires), the infection rate was 5.0% (10/199).

### Microbiology and management

Microbiological data were reported in only a minority of studies. When available, *Staphylococcus aureus* (MSSA and MRSA) was the most frequent isolate, while occasional reports described coagulase-negative staphylococci and Gram-negative bacilli. However, more than two-thirds of the included articles did not specify culture results.

Open fractures (*n* = 228) were generally managed with perioperative first- or second-generation cephalosporins, sometimes combined with aminoglycosides for Gustilo type III injuries. However, very few studies stratified infection rates by open versus closed fractures, limiting reliable comparisons.

Management strategies varied by infection severity: superficial SSI and most pin-tract infections were treated conservatively with local wound care and oral antibiotics; deep SSI/FRI typically required intravenous antibiotics with surgical debridement. Among the 71 reoperations, hardware was removed in cases of instability or persistent infection, retained temporarily if fracture healing was sufficient or near completion, or exchanged in staged procedures. Timing of reoperation was variably reported, but generally occurred within the first 3 months.

## Discussion

The present systematic review synthesizes evidence from 55 clinical studies including 6499 patients undergoing 6451 procedures for distal radius fractures (DRFs). An overall infection rate of 5.3% was observed, with clear variability across fixation methods: 2.0% after ORIF, 12.2% after Kirschner-wire fixation, and 13.9% after external fixation. These results highlight that although infections remain relatively uncommon, their frequency differs substantially according to the chosen surgical technique.

The predominance of pin-tract infections in percutaneous and external fixation constructs (61% of reported cases) is consistent with prior single-center series and randomized trials [10, 24, 32, 36, 37, 44, 49, 50, 52–55, 57, 59, 62, 64, 65, 67–69, 71, 74]. Such infections were mostly minor, responded to local care and short antibiotic courses, and rarely necessitated hardware removal. Conversely, deep SSI and FRI—mainly associated with plate fixation or staged conversion procedures—required intravenous therapy and surgical revision, often with debridement and hardware removal [21, 27, 38, 41, 42, 56, 62, 68, 75]. These findings align with evidence from prospective cohorts where deep infection after ORIF, although rare, carried significant morbidity, sometimes leading to malunion or reoperation [33–35, 42, 43, 46, 48, 56].

A critical methodological issue concerns the heterogeneous definitions of infection across studies. Some authors reported “superficial” versus “deep” infection [33–35, 46, 47], whereas others only mentioned “wound complications” or “pin-site problems” without applying standardized criteria [8, 20, 22, 23, 30, 36, 37, 44]. According to CDC guidelines, SSI are limited to 30 days (90 days in the presence of implants), whereas recent consensus statements recommend using the term fracture-related infection (FRI) for implant-associated cases up to 12 months postoperatively [7]. Most included studies predated or did not adopt these consensus definitions, contributing to variability. Our review therefore distinguished SSI, FRI, and pin-tract infections separately, in line with current recommendations.

Microbiological reporting was limited. Only a minority of studies provided culture results [21, 38, 41, 45, 56, 68], with *Staphylococcus aureus* (both MSSA and MRSA) being the most frequent isolate, followed by occasional coagulase-negative staphylococci and Gram-negative organisms [43, 52]. The scarcity of data precludes reliable conclusions on pathogen distribution or resistance patterns. This represents an important gap, since targeted therapy and prophylaxis strategies depend on robust microbiological evidence.

The role of open fractures remains difficult to quantify. A total of 228 open injuries were identified across studies [41–43, 49, 54, 59, 66], but very few stratified infection outcomes specifically for open versus closed fractures. Most authors reported standard prophylaxis with first- or second-generation cephalosporins, sometimes supplemented with aminoglycosides in Gustilo type III cases [41, 42, 49, 54]. Although infection appeared more common in cohorts with higher proportions of open fractures [43, 49], insufficient stratification prevented pooled estimates. Future studies should systematically differentiate infection rates in open and closed injuries.

Reoperations occurred in at least 71 cases, usually for deep SSI/FRI following ORIF. Where detailed, management included hardware removal when unstable or persistently infected [21, 27, 38, 42, 56], retention when fracture healing was sufficient [32, 41, 68], or staged exchange procedures [62]. Timing was variably reported, but most occurred within three postoperative months [21, 38, 41, 42, 56, 68]. These findings emphasize that although uncommon, deep infections can substantially complicate the postoperative course.

When comparing our results to previous reviews of DRF fixation, infection has typically been reported as a secondary endpoint, often pooled under “complications” alongside loss of reduction or tendon irritation [13–17, 76, 77]. To our knowledge, no prior review has systematically focused on the epidemiology, classification, microbiology, and treatment of DRF surgery. Our synthesis therefore provides novel insights by collating treatment-stratified infection rates across a large patient population.

## Limits of the study

Several limitations must be acknowledged. First, the overall evidence base is heterogeneous: most included studies were retrospective and not primarily designed to evaluate infection. Second, definitions of infection varied considerably, and reporting of microbiology and antibiotic regimens was sparse. Third, although we applied rigorous PRISMA methodology and MINORS quality assessment, no meta-analysis was feasible due to variability in outcome definitions and incomplete stratification by fracture type or fixation method.

Despite these limitations, our findings have practical implications. Pin-tract infections should be anticipated and minimized through meticulous pin-site care in EF and K-wire fixation, while ORIF, although carrying a lower infection rate, may lead to more severe complications requiring surgical revision. Smoking cessation, careful handling of open fractures, and timely conversion from temporary to definitive fixation may reduce infection risk [21, 24, 27, 42, 43, 49, 68]. More consistent adoption of standardized SSI/FRI definitions and systematic microbiological reporting are essential to improve comparability across studies and to guide evidence-based preventive and therapeutic strategies.

## Conclusions

Infections following surgical treatment of distal radius fractures are relatively uncommon but show substantial variability across fixation methods. Pin-tract infections predominate in percutaneous and external fixation, whereas deep SSI/FRI are infrequent after ORIF but associated with greater morbidity and need for reoperation. The scarcity of microbiological data and inconsistent definitions across studies limit comparability and evidence-based recommendations. Standardized classification of SSI versus FRI, systematic microbiological reporting, and dedicated analyses of open fractures are essential to advance prevention and treatment strategies. Clinically, meticulous pin-site care, appropriate perioperative antibiotic prophylaxis, and timely management of open injuries remain key to reducing infection risk.

## Supplementary Information

Below is the link to the electronic supplementary material.

Supplementary Material 1

## Data Availability

No datasets were generated or analysed during the current study.

## Abbreviations

DRF: Distal radius fracture
ORIF: Open reduction and internal fixation
EF: External fixation
CRPS: Complex regional pain syndrome
AO: Arbeitsgemeinschaft für Osteosynthesefragen
mm: Millimeters
°: Degrees
PRISMA: Preferred Reporting Items for Systematic Reviews and Meta-Analyses
MINORS: Methodological Index for Non-Randomized Studies
SPSS: Statistical Package for the Social Sciences
SSI: Surgical Site Infection
CDC: Centers for Disease Control and Prevention
MRSA: Methicillin-resistant Staphylococcus aureus
MSSA: Methicillin-sensitive Staphylococcus aureus
RCT: Randomized Controlled Trial
ASA: American Society of Anesthesiologists
ED: Emergency Department

## References

[1] Rundgren J, Bojan A, Mellstrand Navarro C, Enocson A (2020) Epidemiology, classification, treatment and mortality of distal radius fractures in adults: an observational study of 23,394 fractures from the National Swedish fracture register. BMC Musculoskelet Disord 21:88. 10.1186/s12891-020-3105-1 32035488 10.1186/s12891-020-3097-8 PMC7007648

[2] Azad A, Kang HP, Alluri RK, Vakhshori V, Kay HF, Ghiassi A et al (2019) Epidemiological and treatment trends of distal radius fractures across multiple age groups. J Wrist Surg 8:305–311. 10.1055/s-0039-1688504 31404224 10.1055/s-0039-1685205 PMC6685779

[3] Corsino CB, Reeves RA, Sieg RN (2024) Distal radius fractures. StatPearls. StatPearls Publishing, Treasure Island (FL) 30725601

[4] Chung KC, Shauver MJ, Birkmeyer JD (2011) Variations in the use of internal fixation for distal radial fracture in the united States medicare population. J Bone Joint Surg Am 93:2154–2162. 10.2106/JBJS.J.00961 22159850 10.2106/JBJS.J.012802 PMC3226419

[5] Koval KJ, Harrast JJ, Anglen JO, Weinstein JN (2008) Fractures of the distal part of the radius: the evolution of practice over time. Where’s the evidence? J Bone Joint Surg Am 90:1855–1861. 10.2106/JBJS.H.00379 18762644 10.2106/JBJS.G.01569

[6] Rundgren J, Bojan A, Mellstrand Navarro C, Enocson A (2020) Surgical site infections after distal radius fracture surgery: a nationwide cohort study of 31,807 adult patients. BMC Musculoskelet Disord 21:845. 10.1186/s12891-020-03846-0 33339519 10.1186/s12891-020-03822-0 PMC7749509

[7] Metsemakers WJ, Morgenstern M, McNally MA, Moriarty TF, McFadyen I, Scarborough M et al (2018) Infection after fracture fixation: current surgical and microbiological concepts. Injury 49:511–522. 10.1016/j.injury.2016.09.019 27639601 10.1016/j.injury.2016.09.019

[8] Kong L, Liu Y, Zhou M, Yu M, Zhang J, Zhang J et al (2024) Factors predicting complications following open reduction and internal fixation of intra-articular distal radius fracture. Front Surg 11:1356121. 10.3389/fsurg.2024.1356121 38586239 10.3389/fsurg.2024.1356121 PMC10998443

[9] Meng H, Xu J, Liu J, Zhang C, Yang Y, Wang J (2022) Incidence and risk factors for surgical site infection following volar locking plating of unstable distal radius fracture. J Orthop Surg Res 17:549. 10.1186/s13018-022-03271-2 36529774 10.1186/s13018-022-03440-7 PMC9762064

[10] Egol KA, Walsh M, Tejwani NC, McLaurin T, Wynn C, Paksima N (2006) Treatment of external fixation pins about the wrist: a prospective, randomized trial. J Bone Joint Surg Am 88:349–354. 10.2106/JBJS.E.00102 16452747 10.2106/JBJS.E.00011

[11] Berríos-Torres SI, Umscheid CA, Bratzler DW, Leas B, Stone EC, Kelz RR et al (2017) Centers for disease control and prevention guideline for the prevention of surgical site infection, 2017. JAMA Surg 152:784–791. 10.1001/jamasurg.2017.0904 28467526 10.1001/jamasurg.2017.0904

[12] Metsemakers WJ, Moriarty TF, Nijs S, Pape HC, Richards RG, Verhofstad MHJ et al (2016) Infection after fracture fixation: current surgical and Microbiological concepts. Injury 47:302–308. 10.1016/j.injury.2016.04.019 10.1016/j.injury.2016.09.019 27639601

[13] Mauck BM, Swigler CW (2018) Evidence-based review of distal radius fractures. Orthop Clin North Am 49:211–222. 10.1016/j.ocl.2018.01.001 29499822 10.1016/j.ocl.2017.12.001

[14] Gehrmann SV, Windolf J, Kaufmann RA (2008) Distal radius fracture management in elderly patients: a literature review. J Hand Surg Am 33:421–429. 10.1016/j.jhsa.2008.01.026 18343302 10.1016/j.jhsa.2007.12.016

[15] Karantana A, Downing ND, Forward DP, Hatton M, Taylor AM, Scammell BE et al (2013) Surgical treatment of distal radial fractures with a volar locking plate versus conventional percutaneous methods: a randomized controlled trial. J Bone Joint Surg Am 95:1737–1744. 10.2106/JBJS.L.00232 24088965 10.2106/JBJS.L.00232

[16] Costa ML, Achten J, Plant C, Parsons NR, Rangan A, Lamb SE et al (2015) UK DRAFFT: a randomised controlled trial of percutaneous fixation with Kirschner wires versus volar locking-plate fixation in the treatment of adult patients with a dorsally displaced fracture of the distal radius. Health Technol Assess 19:1–124. 10.3310/hta19170 25716883 10.3310/hta19170 PMC4781149

[17] Chaudhry H, Kleinlugtenbelt YV, Mundi R, Ristevski B, Goslings JC, Bhandari M (2015) Are volar locking plates superior to percutaneous K-wires for distal radius fractures? A meta-analysis. Clin Orthop Relat Res 473:3017–3027. 10.1007/s11999-015-4253-6 25981715 10.1007/s11999-015-4347-1 PMC4523532

[18] Page MJ, McKenzie JE, Bossuyt PM, Boutron I, Hoffmann TC, Mulrow CD et al (2021) The PRISMA 2020 statement: an updated guideline for reporting systematic reviews. BMJ 372:n71. 10.1136/bmj.n71 33782057 10.1136/bmj.n71 PMC8005924

[19] Slim K, Nini E, Forestier D, Kwiatkowski F, Panis Y, Chipponi J (2003) Methodological index for non-randomized studies (MINORS): development and validation of a new instrument. ANZ J Surg 73:712–716. 10.1046/j.1445-2197.2003.02748.x 12956787 10.1046/j.1445-2197.2003.02748.x

[20] Huang X, Wang Y, Yang H, Zhang J, Zhang Y (2023) Evaluation of the treatment of distal radial volar fracture by different methods sparing the pronator quadratus. J Orthop Surg Res 18:722. 10.1186/s13018-023-04184-8 37749563 10.1186/s13018-023-04184-8 PMC10519083

[21] Ficke B, Jafari M, Faruqui S, Hill JR (2018) Outcomes of staged treatment for complex distal radius fractures. Cureus 10:e3273. 10.7759/cureus.3273 30443444 10.7759/cureus.3273 PMC6235653

[22] Akdemir M, Erdoğan M, Kömürcü M, Tunçer M, Çabuk H, Kacira B et al (2024) Open reduction and plate fixation, external fixator, and conservative treatment for intra-articular distal radius fractures. Cureus 16:e52014. 10.7759/cureus.52014 38344567 10.7759/cureus.52014 PMC10854372

[23] Crook JL, Pientka W, Zhang AY, Golden A, Koehler D, Sammer D (2024) Risk factors for surgical site infection after surgical treatment of closed distal radial fractures. J Hand Surg Eur 49:310–315. 10.1177/17531934231194672 10.1177/17531934231194672 37666217

[24] Wasiak M, Grudziak J, Moszura T, Domzalski M (2023) Early complications of percutaneous K-wire fixation in pediatric distal radius fractures: a prospective cohort study. Arch Orthop Trauma Surg 143:6649–6656. 10.1007/s00402-023-04846-9 37522939 10.1007/s00402-023-04996-7 PMC10541837

[25] Kotsalis G, Giannakopoulos N, Fyllos A, Chatzopoulos S, Xarchas K, Kelekis A et al (2023) Three column fixation through a single incision in distal radius fractures. J Wrist Surg 12:232–238. 10.1055/s-0042-1749210 37223379 10.1055/s-0042-1749162 PMC10202585

[26] Abdullah S, Rijal L, Nather A, Shukla M, Masri BA (2023) A prospective study comparing the infection rate between buried vs exposed Kirschner wires in hand and wrist fixations. Cureus 15:e36558. 10.7759/cureus.36558 37102015 10.7759/cureus.36558 PMC10123197

[27] Eckstein C, Pässler T, Hanke M, Pape HC, Keller J (2023) Operative treatment of distal radial fractures under vitamin K antagonist or DOAC: is preoperative interruption of these drugs necessary? Unfallchirurgie 126:463–467. 10.1007/s00113-023-01287-1 37014375 10.1007/s00113-023-01311-2

[28] Moutinot B, Durand S, Maillot C, Dap F, Dautel G (2023) Perioperative morbidities in distal radius fractures treated using locking plates in the super-elderly population: a retrospective study. J Hand Surg Glob Online 5:140–144. 10.1016/j.jhsg.2022.11.006 36974297 10.1016/j.jhsg.2022.11.004 PMC10039287

[29] Zhu Y, Zhang C, Xu J, Meng H, Cheng T, Wang J et al (2022) Risk factors for complications following volar locking plate fixation of unstable distal radius fracture. Biomed Res Int 2022:9117533. 10.1155/2022/9117533 36483632 10.1155/2022/9117533 PMC9726249

[30] Meng H, Wang Y, Xu J, Cheng T, Wang J (2022) Treatment of distal radius fractures using a cemented K-wire frame. BMC Musculoskelet Disord 23:591. 10.1186/s12891-022-05603-w 35725465 10.1186/s12891-022-05550-z PMC9208138

[31] Zhong J, Chen J, Zhang C, Han S, Zhu Y (2022) Clinical applications of internal fixation via the volar approach with pronator quadratus preservation for distal radius fractures. Turk J Med Sci 52:1177–1182. 10.3906/sag-2112-93 36326410 10.55730/1300-0144.5421 PMC10387832

[32] Chung KC, Malay S, Shauver MJ, Kim HM, WRIST Group (2019) Assessment of distal radius fracture complications among adults 60 years or older: a secondary analysis of the WRIST randomized clinical trial. JAMA Netw Open 2:e187053. 10.1001/jamanetworkopen.2018.7053 30657531 10.1001/jamanetworkopen.2018.7053 PMC6484535

[33] Jose A, Babu A, Reddy S, Bhat A, Shetty A, Nair P (2017) Unstable distal radius fractures treated by volar locking anatomical plates. J Clin Diagn Res 11:RC04–RC08. 10.7860/JCDR/2017/22746.9252 28274009 10.7860/JCDR/2017/24114.9261 PMC5324454

[34] Khatri K, Sharma V, Farooque K, Tiwari A, Sharma S (2016) Surgical treatment of unstable distal radius fractures with a volar variable-angle locking plate: clinical and radiological outcomes. Arch Trauma Res 5:e25174. 10.5812/atr.25174 27679785 10.5812/atr.25174 PMC5035514

[35] Natoli RM, Baer MR, Bednar MS (2016) Conversion of external fixation to open reduction and internal fixation for complex distal radius fractures. Orthop Traumatol Surg Res 102:339–343. 10.1016/j.otsr.2016.01.005 27026500 10.1016/j.otsr.2016.01.013

[36] Chilakamary VK, Naidu B, Reddy YK, Vemula R (2016) Osteosynthesis in distal radius fractures with conventional bridging external fixator: tips and tricks for getting them right. J Clin Diagn Res 10:RC05–RC08. 10.7860/JCDR/2016/16747.7216 26894133 10.7860/JCDR/2016/16696.7048 PMC4740661

[37] Mirghasemi SA, Ramezan Shirazi M, Saied AR, Karimi A (2015) A prospective study of a modified pin-in-plaster technique for treatment of fractures of the distal radius. Bone Joint Res 4:176–180. 10.1302/2046-3758.411.2000482 26541833 10.1302/2046-3758.411.2000429 PMC4649681

[38] Matullo KS, Dennison DG (2015) Outcome following distally locked volar plating for distal radius fractures with metadiaphyseal involvement. Hand (NY). 10:292–296. 10.1007/s11552-014-9683-2 10.1007/s11552-014-9713-z PMC4447681 26034446

[39] Herzberg G, Walch A, Burnier M (2018) Wrist hemiarthroplasty for irreparable distal radius fractures in the elderly. Eur J Orthop Surg Traumatol 28:1499–1503. 10.1007/s00590-018-2202-5 29796826 10.1007/s00590-018-2228-5

[40] Ozkan S, Coşkunol E, Akpinar S, Ozkan K (2018) Distal radius fractures: evaluation of closed reduction and percutaneous Kirschner wire pinning. J Hand Microsurg 10:134–138. 10.1055/s-0037-1618570 30483019 10.1055/s-0038-1648334 PMC6255731

[41] Takada N, Otsuka T, Yonekura A, Saito K, Kamogawa J (2012) Minimally invasive plate osteosynthesis for distal radius fractures with a palmar locking plate. Eur J Trauma Emerg Surg 38:627–632. 10.1007/s00068-011-0164-3 26814548 10.1007/s00068-012-0204-z

[42] Tarallo L, Mugnai R, Adani R, Catani F (2013) Volar plate fixation for the treatment of distal radius fractures: analysis of adverse events. J Orthop Trauma 27:740–745. 10.1097/BOT.0b013e31828c6a8a 23515129 10.1097/BOT.0b013e3182913fc5

[43] Kaufman AM, Bae DS, Rizzo M, Sammer DM (2014) Safety of immediate open reduction and internal fixation of geriatric open fractures of the distal radius. Injury 45:534–539. 10.1016/j.injury.2013.10.028 24262670 10.1016/j.injury.2013.10.006

[44] Gogna P, Selhi HS, Mohindra M, Singla R, Kalra M, Yamin M (2013) Osteosynthesis with long volar locking plates for metaphyseal-diaphyseal fractures of the distal radius. Chin J Traumatol 16:339–343. 10.3760/cma.j.issn.1008-1275.2013.06.002 24295579

[45] Wei XM, Song CJ, Jin LJ (2014) Minimally invasive plate osteosynthesis for distal radius fractures. Indian J Orthop 48:20–24. 10.4103/0019-5413.125483 24600058 10.4103/0019-5413.125483 PMC3931148

[46] Lauder A, Hanel DP, Chapman TR, Huang JI (2015) Functional outcomes following bridge plate fixation for distal radius fractures. J Hand Surg Am 40:1554–1562. 10.1016/j.jhsa.2015.05.021 26143028 10.1016/j.jhsa.2015.05.008

[47] Das AK, Sundaram N, Devadoss S, Ravichandran S (2011) Percutaneous pinning for non-comminuted extra-articular fractures of distal radius. Indian J Orthop 45:422–426. 10.4103/0019-5413.82335 21886923 10.4103/0019-5413.83949 PMC3162678

[48] Kurylo JC, Axelrad TW, Tornetta P 3rd, O’Toole RV, Nascone JW, Sciadini MF et al (2011) Open fractures of the distal radius: the effects of delayed debridement and immediate internal fixation on infection rates and the need for secondary procedures. J Hand Surg Am 36:1131–1134. 10.1016/j.jhsa.2011.04.025 21636223 10.1016/j.jhsa.2011.04.014

[49] Lakshmanan P, Matthewson G, Young C (2010) Infection rate of percutaneous Kirschner wire fixation for distal radius fractures. J Orthop Surg (Hong Kong) 18:85–86. 10.1177/230949901001800118 20427842 10.1177/230949901001800119

[50] Wick M, Wiesner M (2010) Mid-term results after volar plating of distal radius fractures with a newly designed locking plate. Z Orthop Unfall 148:66–71. 10.1055/s-0029-1240969 20135602 10.1055/s-0029-1186203

[51] Tyllianakis M, Panagopoulos A, Papadopoulos AX, Papasimos S, Mousafiris K (2010) Treatment of unstable distal radius fractures with Ilizarov circular, nonbridging external fixator. Injury 41:306–311. 10.1016/j.injury.2009.09.013 20176171 10.1016/j.injury.2009.09.011

[52] Herdrich S, Kuhn S, Rommens PM (2010) Management of complex intra-articular distal radius fractures with open reduction and internal fixation with double dorsal locking plates. Z Orthop Unfall 148:72–79. 10.1055/s-0029-1240983 20135600 10.1055/s-0029-1186156

[53] Zettl RP, Clauberg E, Klos K, Gras F, Hofmann GO (2009) Volar locking compression plating versus dorsal plating for fractures of the distal radius: a prospective, randomized study. Unfallchirurg 112:712–718. 10.1007/s00113-009-1669-4 19597773 10.1007/s00113-008-1526-5

[54] Glueck DA, Charoglu CP, Lawton JN (2009) Factors associated with infection following open distal radius fractures. Hand (N Y) 4:330–334. 10.1007/s11552-009-9170-0 19194762 10.1007/s11552-009-9173-z PMC2724624

[55] Pieske O, Kalicke T, Zettl R, Trentzsch H, Piltz S, Matschke S et al (2008) Titanium alloy pins versus stainless steel pins in external fixation at the wrist: a randomized prospective study. J Trauma 64:1275–1280. 10.1097/TA.0b013e31806dc48d 18469650 10.1097/TA.0b013e31815e40e0

[56] Strohm PC, Müller CA, Boll T, Pfister U (2007) Ist die winkelstabile, palmare 3,5-mm-T-Platte die Lösung für dislozierte, distale radiusfrakturen?? Z Orthop Unfall 145:331–337. 10.1055/s-2007-965039 17607633 10.1055/s-2007-965348

[57] Wong KK, Chan KW, Kwok TK, Mak KH (2005) Volar fixation of dorsally displaced distal radial fracture using locking compression plate. J Orthop Surg (Hong Kong) 13:153–157. 10.1177/230949900501300210 16131677 10.1177/230949900501300208

[58] Lee HC, Wong YS, Chan BK, Low CO (2003) Fixation of distal radius fractures using AO titanium volar distal radius plate. Hand Surg 8:7–15. 10.1142/S0218810403001406 12923928 10.1142/s0218810403001339

[59] Ahlborg HG, Josefsson PO (1999) Pin-tract complications in external fixation of fractures of the distal radius. Acta Orthop Scand 70:116–118. 10.3109/17453679908997802 10366908 10.3109/17453679909011246

[60] Chin KR, Jupiter JB (1999) Wire-loop fixation of volar displaced osteochondral fractures of the distal radius. J Hand Surg Am 24:525–533. 10.1053/jhsu.1999.0525 10357531 10.1053/jhsu.1999.0525

[61] Fritz EM, Donato DP, Westberg JR, Geissler JA, Ward CM (2024) Incidence of infection in operatively treated distal radius fractures after conversion from external to internal fixation. J Hand Surg Am 49:184.e1–184.e7. 10.1016/j.jhsa.2022.06.009 10.1016/j.jhsa.2022.06.009 35931630

[62] Lundqvist E, Hammer R, Wennergren D, Rosberg HE, Brogren E (2022) Volar locking plate compared with combined plating of AO type C distal radius fractures: a randomized controlled study of 150 cases. J Hand Surg Am 47:813–822. 10.1016/j.jhsa.2022.04.013 35842329 10.1016/j.jhsa.2022.04.018

[63] Ahmad F, Gardner B, Blagg S, Karantana A (2022) Does time to operative intervention of distal radius fractures influence outcomes? Hand (N Y). 17:135S–139S. 10.1177/15589447221114794 10.1177/15589447211072219 PMC9793627 35695167

[64] Michael G, Dasari V, Mahapatra A, Sharma R (2022) Functional outcome of joshi’s external stabilization system fixation in distal radius fractures. Cureus 14:e24215. 10.7759/cureus.24215 35602785 10.7759/cureus.24215 PMC9117850

[65] Liechti R, Beeres FJP, Kach K, Wanner GA, Simmen HP, Seiler C et al (2022) The spanning plate as an internal fixator in complex distal radius fractures: a prospective cohort study. Eur J Trauma Emerg Surg 48:2369–2377. 10.1007/s00068-021-01762-2 34185106 10.1007/s00068-021-01738-5

[66] Henry TW, Ruchelsman DE, Lee SK (2022) Outcomes of type I open distal radius fractures: a comparison of delayed and urgent open reduction internal fixation. Hand (N Y) 17:952–956. 10.1177/15589447211019041 33215540 10.1177/1558944720964965 PMC9465772

[67] Gaibor GG, Tello CA, Gaibor AG (2020) Avaliação radiográfica Dos Pacientes submetidos a Fixação percutânea com Parafuso Maciço Para Tratamento de fraturas Da extremidade distal do rádio. Rev Bras Ortop 55:605–611. 10.1055/s-0039-3402777

[68] Maleitzke T, Clausen JD, Wichlas F, Greiner A, Märdian S (2020) Haematoma block: a safe method for pre-surgical reduction of distal radius fractures. J Orthop Surg Res 15:351. 10.1186/s13018-020-01914-6 32843043 10.1186/s13018-020-01819-y PMC7448324

[69] Maradei-Pereira JA, Carazzato JG, De Rezende MR, Mattar R Jr. (2021) Infection after buried or exposed K-wire fixation of distal radial fractures: a randomized clinical trial. J Hand Surg Eur 46:154–158. 10.1177/1753193420958865 10.1177/1753193420936543 32611274

[70] Tarallo L, Mugnai R, Rocchi M, Adani R, Catani F (2020) Volar PEEK plate for distal radius fracture: analysis of adverse events. Eur J Orthop Surg Traumatol 30:1293–1298. 10.1007/s00590-020-02669-1 32435847 10.1007/s00590-020-02701-7

[71] Liu Y, Bai YM (2020) Efficacy of non-bridging external fixation in treating distal radius fractures. Orthop Surg 12:776–783. 10.1111/os.12682 32343053 10.1111/os.12677 PMC7307264

[72] Kim PH, Leopold SS (2012) In brief: Gustilo-Anderson classification. Clin Orthop Relat Res 470:3270–3274. 10.1007/s11999-012-2376-6 22569719 10.1007/s11999-012-2376-6 PMC3462875

[73] Metsemakers WJ et al (2022) Fracture-related infection: a consensus on definition from an international expert group. Injury 53:361–366. 10.1016/j.injury.2021.10.037 10.1016/j.injury.2017.08.040 28867644

[74] Wasiak M, Gajewski M, Mikusek G, Kołodziej Ł, Bursiewicz S, Janowicz K (2023) Early complications of percutaneous K-wire fixation in pediatric distal radius fractures: a prospective cohort study. Arch Orthop Trauma Surg 143:6649–6656. 10.1007/s00402-023-04927-2 37522939 10.1007/s00402-023-04996-7 PMC10541837

[75] Meng H, Zhuang Y, Luo CF (2022) Incidence and risk factors for surgical site infection following volar locking plating of unstable distal radius fracture. J Orthop Surg Res 17:549. 10.1186/s13018-022-03320-3 36529774 10.1186/s13018-022-03440-7 PMC9762064

[76] Mathews AL, Chung KC (2015) Management of complications of distal radius fractures. Hand Clin 31:205–215. 10.1016/j.hcl.2015.01.011 25934197 10.1016/j.hcl.2014.12.002 PMC4417479

[77] Floquet A, Chanlalit C, Auquit-Auckbur I, Dap F, Dautel G (2021) Flexor tendon rupture after volar plating of distal radius fracture: a systematic review of the literature. Hand Surg Rehabil 40:535–546. 10.1016/j.hansur.2021.04.004 34033928 10.1016/j.hansur.2021.05.008

