# Supplementary material for: Epidemiology and treatment of surgical infections after distal radius fractures: a systematic review
Source: Arch Orthop Trauma Surg. 2025 Oct 23;145(1):483. doi: 10.1007/s00402-025-06061-x (PMC12549420; doi:10.1007/s00402-025-06061-x)
Supplement: Supplementary file 1 — Supplementary Material 1 [file 402_2025_6061_MOESM1_ESM.pdf]

Appendix 1 – Search strategy

PubMed/MEDLINE (searched up to June 30, 2024)

("Radius Fractures" OR "distal radius fracture"[Title/Abstract] OR "DRF"[Title/Abstract] OR "wrist fracture"[Title/Abstract])
AND
("Surgical Wound Infection" OR "surgical site infection"[Title/Abstract] OR "SSI"[Title/Abstract] OR "fracture-related infection"[Title/Abstract] OR "infection"[Title/Abstract])
AND
("Fracture Fixation, Internal" OR "open reduction internal fixation"[Title/Abstract] OR "ORIF"[Title/Abstract] OR "External Fixators" OR "external fixation"[Title/Abstract] OR "Kirschner wire"[Title/Abstract] OR "K-wire"[Title/Abstract])

Cochrane Library (searched up to June 30, 2024)

(distal radius fracture OR DRF OR wrist fracture)
AND
(surgical site infection OR SSI OR fracture-related infection OR infection)
AND
(open reduction internal fixation OR ORIF OR external fixation OR Kirschner wire OR K-wire)
